# Relational Climate in the Workplace: Dimensions, Measurement, and Validation

**DOI:** 10.3389/fpsyg.2020.00085

**Published:** 2020-02-13

**Authors:** Richard E. Boyatzis, Kylie Rochford

**Affiliations:** ^1^Organizational Behavior Department, Case Western Reserve University, Cleveland, OH, United States; ^2^Department of Management, The University of Utah, Salt Lake City, UT, United States

**Keywords:** workplace relationships, relational climate, scale development organizational behavior and human performance, shared vision, shared compassion, shared energy

## Abstract

Relationships are the fundamental building blocks of organizations, yet the field lacks a validated and comprehensive measure of how employees perceive the quality of the relationships in their organization. In this paper, we develop and validate a scale to measure the perceived relational climate in an organization. We operationalize relational climate as a second-order latent construct reflected by three first-order constructs: shared vision, compassion, and relational energy. In Study 1, we develop an item pool consisting of 51 items and then use a Q-sort procedure to assess content validity. In Study 2, the item pool is further reduced using exploratory factor analysis. This is followed by a confirmatory factor analysis that finds initial support for the three-dimensional structure of relational climate. Study 3 provides further evidence of convergent and discriminant validity and assesses the criterion validity of the construct in relation to leader–member social exchange (LMSX), perceived organizational support, and procedural justice (all positive relationships). Finally, in Study 4, the factor structure of the quality-of-relationships scale is successfully replicated, and criterion validity is further assessed in relation to instrumental ethical climate (negative relationship) and affective organizational commitment (positive relationship). This paper contributes a new validated measure to the literature that will allow organizations to capture an important aspect of their work environment—the nature of the interpersonal relationships. Implications for theory, limitations, and future research are discussed.

## Introduction

The importance of high-quality relationships in the workplace is agreed among organizational scholars (Dutton and Ragins, 2007; Heaphy et al., 2018). Benefits of high-quality relationships span multiple levels: from individual benefits such as enhanced psychological well-being (Reis and Gable, 2002) and physical health (Uchino et al., 1996; Berscheid, 1999) to organizational benefits such as enhanced job performance (Gittell et al., 2010), learning (Carmeli et al., 2009), coordination (Gittel, 2003), and error detection (Weick and Roberts, 1993). However, despite the host of benefits associated with high-quality relationships, the field is still lacking a comprehensive and validated measure that captures the overall nature of the relationships in a given environment.

Relational climate is a *facet-specific climate* defined as “shared employee perceptions and appraisals of policies, practices, and behaviors affecting interpersonal relationships in a given context…” (Mossholder et al., 2011: 36). In conceptualizing relational climate, Mossholder and colleagues integrate the structuralist and social interactionist approaches to climate. Specifically, these authors draw on structuralism to argue that relational climate begins with employees’ subjective experiences of the structural aspects of the organization designed to impact interpersonal relationships (i.e., HR policies and practices). They then draw on social interactionism (Berger and Luckmann, 1967) to highlight the role of collective sensemaking processes that facilitate the emergence of shared meanings and perceptions regarding the structural aspects (Weick, 1979). While Mossholder and colleagues carefully laid the theoretical underpinnings of relational climate, to our knowledge, the construct has not yet been operationalized and empirically validated. In this paper, we build on these theoretical foundations to develop and validate a measure of relational climate. In the following section, we outline the theoretical underpinnings of relational climate as a latent construct that can be measured indirectly by the perceived degree of (1) shared vision, (2) compassion, (3) positive mood, and (4) relational energy in a given dyadic relationship, team, or organization.

## Operational Definition

Mossholder and colleagues draw on Fiske’s (1992) relational models theory to identify difference characterizations of relational climate: market pricing, equality matching, and communal sharing. These models represent different “types” of relationship that reflect different motivations and rules for interrelating. In considering how best to operationalize relational climate, we depart slightly, but in a compatible way, from Mossholder et al. (2011) conceptualization of relational climate by incorporating the growing literature in the domain of positive organizational scholarship. In doing so, we focus primarily on measuring the extent to which people perceive the relational climate in their organization to reflect “high-quality” relationships. High-quality relationships are “manifested in shared goals, shared knowledge, and mutual respect” (Carmeli and Gittell, 2009: 714). Relating this to Mossholder et al. (2011) different types of relational climate, we are primarily focusing our efforts on operationalizing a relational climate that reflects the communal sharing model of relating (see Fiske, 1992, and Mossholder et al., 2011), which is characterized by relationships that are based on shared values, affective bonds, and empathetic concern (Mossholder et al., 2011).

Building off the definition of high-quality relationships and in line with the communal sharing model of relationships (Fiske, 1992), in this paper, we propose that relational climate can be operationalized by four first-order latent constructs: the degree of shared vision, compassion, relational energy, and positive mood in a given environment. In what follows, we provide a theoretical rationale for each of the four proposed factors. We then present the results of a full-scale development process as outlined by Hinkin (1998), resulting in a psychometrically sound and validated measure of relational climate.

### Definitions and Theoretical Rationale for First-Order Factors

#### Shared Vision

Shared vision is defined as “the extent to which members of an organization (or team or dyad) share a common mental image of a desirable future that provides a basis for action” (adapted from Pearce and Ensley, 2004: 260–261). The role of shared vision in relational climate is 2-fold. First, in the context of the workplace, shared vision creates a sense of belonging, social identity, and internalization of values and attitudes—all of which are characteristics of high-quality relationships (Kahn, 2007) and are also consistent with Fiske’s (1992) notion of communal sharing and thus Mossholder et al. (2011) communal sharing model of relational climate. Second, the presence of a shared vision suggests that the relationships incorporated within a given dyad, group, or team have moved beyond our initial basic needs to be liked and to belong (Baumeister and Leary, 1995) to more profound, meaningful, and sustainable bonds (Allport, 1962).

#### Compassion

We adopt Boyatzis et al. (2012) definition of compassion as “an interpersonal process that involves noticing another person as being in need, empathizing with him or her, and acting to enhance his or her well-being in response to that need” (pp. 154–155). This definition departs slightly from the traditional view of compassion in that it replaces the term “suffering,” which, by definition, requires pain, distress, or hardship, with the broader term of “being in need,” which allows for both noticing and acting not only to ease a person’s suffering but also to enhance their subjective and psychological well-being. Consistent with mainstream literature, compassion is conceptualized as consisting of three components: (a) noticing or attending to another’s need; (b) other-regard feelings such as empathic concern; and (c) acting to ease the suffering and/or enhance well-being (Kanov et al., 2004; Dutton et al., 2006). Compassion is a mechanism that facilitates a sense of value and worth in interactions (Frost, 2003; Dutton et al., 2014) and creates a psychological pull between people that strengthens the connection between them (Dutton et al., 2002).

#### Positive Mood

Positive mood is defined as “a transient affective state characterized by feelings of positive emotions such as enthusiasm, elation, and excitement that are not focused on any particular object, event, individual, or behavior” (adapted from Watson et al., 1988, and George and Brief, 1992). In this paper, we conceptualize positive mood as a state and therefore as distinct from generalized positive affect and similar constructs including optimism and positive outlook (Watson and Pennebaker, 1989). We contend that when we interact with others in a meaningful and positive way, the resulting affective response is reflective of positive mood. Thus, one reflection of the nature of the relationship/s in a dyad, team, or organization is the degree of positive mood.

#### Relational Energy

The sense of energy that is derived from high-quality relationships is a relatively new idea in the organizational sciences. The concept was introduced into our literature with the rise of the positive psychology movement in the early 2000s by the work of Quinn and Dutton (2005), who claimed that perceived energy in organizations is a result of interactions and, specifically, conversations among individuals. Scholars drew heavily on Ryan and Frederick’s (1997) work on subjective vitality, defined as “a specific psychological experience of possessing enthusiasm and spirit” (p. 530). In this paper, we are particularly interested in energy derived from a person’s relationships in an organization. We draw on the work of Ryan and Frederick (1997) and Quinn and Dutton (2005) to define relational energy as “the extent to which relationships in an organization are a source of energy in that they result in feelings of positive arousal, aliveness, and eagerness to act. Recent research has linked relational energy to perceived social support and the quality of the leader–follower relationship (Owens and Hekman, 2016). For a comprehensive review, see Baker, 2019).

In sum, theory and prior empirical work suggest that relational climate can be operationalized as four first-order latent factors composed of shared vision, compassion, positive mood, and relational energy. Table 1 provides a definition for each construct, the rationale for including it, and citations to relevant supporting literature.

**TABLE 1:** Summary of operational construct of relational climate.

**Construct name**	**Definition**	**Rationale**	**Theory**
Shared vision	The extent to which members of an organization share a common mental image of a desirable future that provides a basis for action.	Identity, belonging, purpose, meaning	Pearce and Ensley, 2004; Kahn, 2007
Compassion	An interpersonal process that involves noticing another person as being in need, empathizing with him or her, and acting to enhance his or her well-being in response to that need.	Caring, feeling valued, psychological connection, mutuality	Miller and Stiver, 1997; Dutton et al., 2014, 2002, 2014; Frost, 2003; Boyatzis et al., 2012
Positive mood	A transient affective state characterized by feelings of positive emotions such as enthusiasm, elation, and excitement that are not focused on any particular object, event, individual, or behavior.	Affective response to positive interactions; attraction to others; broaden and build	Bell, 1978; George and Brief, 1992; Watson et al., 1988; Fredrickson, 2001
Relational energy	The extent to which relationships in an organization are a source of energy in that they result in feelings of positive arousal, aliveness, and eagerness to act.	Energy in organizations is a result of interactions and conversation; only “secure” relationships result in positive energy; energy is contagious.	Ryan and Frederick, 1997; Baker et al., 2003; Quinn and Dutton, 2005; Luke et al., 2012; Owens and Hekman, 2016; Baker, 2019

## Study 1: Construct Definitions and Item Generation

The objective of the first study was to assess the content validity of the proposed item pool. The list of items used in this study was derived deductively due to the strong theoretical foundations that existed for each of the four proposed constructs (Hinkin, 1998). All studies were approved by the Institutional Review Board at Case Western Reserve University (IRB-2015-1067).

### Method

The initial assessment of the content validity of the items involved a small sample of subject matter experts, who were asked to assess the face validity of the proposed items based on the construct definitions and literature. Five doctoral students, ten practitioners, and four faculty members provided detailed feedback on the initial items. As a result of this process, the initial pool of 51 items was developed—14 for shared vision, 13 for compassion, 12 for positive mood, and 12 for relational energy.

We used the approach recommended by Schriesheim et al. (1993) to empirically assess content adequacy. Four separate samples (one per construct) were recruited using Amazon Mechanical Turk (MTurk). MTurk is an online participant pool in which individuals voluntarily participate in advertised studies in return for a small payment (Buhrmester et al., 2011; Goodman et al., 2013). The screening criteria used were as follows: (1) currently living in the United States; (2) being 18 years of age or higher; (3) being able to speak English as their primary language; (4) holding a bachelor’s degree or higher; and (5) having at least 20 accepted HITS (tasks previously completed and submitted on AMT) and an acceptance rate of 95% of previously submitted tasks. Work experience was not required for this study as classifying items is primarily a cognitive task (Schriesheim et al., 1993; Hinkin, 1998). Sample sizes were *N* = 46, *N* = 49, *N* = 49, and *N* = 47. Sociodemographic information for this sample and the samples used in subsequent studies can be found in Table 3.

Participants were given one construct definition along with the full item pool and were asked the extent to which each item corresponds to the construct definition provided. Items were rated on a 5-point Likert scale ranging from 1 = very weak fit to 5 = very strong fit. Three attention checks were included in each survey. Participants who missed more than one of these checks were not included in the sample. To analyze the responses, we calculated a Q-correlation matrix and used principal components analysis. Items that had factor loadings >0.4 (Ford et al., 1986), with no major cross loadings, were retained for Study 2. Items with factors loadings <0.4 or items with major cross loadings were discarded.

### Results and Discussion

Due to space limitations, the full Q-sort results are not displayed, however, they are available on request. As a result of this initial sorting, six items were discarded from the item pool. These items and the reason for discarding them are displayed in Table 2.

**TABLE 2:** Items discarded based on Q-sort results.

**Item**	**Reason for discarding**
My organization is proud of its vision.	Cross loading
Members of my organization have compatible goals.	Cross loading
When we work together, my organization is enthusiastic.	Cross loading
My organization encourages each other to build on our strengths.	Factor loading < 0.40 (β = 0.31)
Generally, people in my organization are relaxed.	Cross loading
In my organization, we emphasize our current strengths.	Factor loading < 0.40 (β = 0.25)

## Study 2: Psychometric Properties of Relational Climate

The objective of Study 2 was to assess the psychometric properties of the proposed relational climate construct. Specifically, the study examined (1) the underlying structure of the latent variable; (2) the reliability of the proposed factors; (3) convergent validity; and (4) discriminant validity.

### Hypotheses

The rationale for operationalizing relational climate as a latent construct that can be measured indirectly by shared vision, compassion, energy, and positive mood is outlined earlier in this paper. In sum, high-quality and positive relationships require a sense of belonging, identity, and shared understanding that, in an organizational setting, can be captured by the degree of shared vision. Compassion creates a sense of being valued and cared for and creates a psychological pull between people (Dutton et al., 2014). Energy and positive mood are indicative of a work environment characterized by secure relationships (Luke et al., 2012) and psychologically safe relationships (Kark and Carmeli, 2009). Thus, we hypothesize the following:

H1a: Relational climate is a second-order latent construct defined by four distinct dimensions of shared vision, compassion, relational energy, and positive mood.

In order for the first-order factors of shared vision, compassion, energy, and positive mood to be meaningful, the observed variables (items) must show evidence of convergent validity with their respective factors. We expect that the observed variables will all be positively correlated with each other, however, each will load significantly on one and only one first-order factor. Similarly, the first-order factors must converge on the higher-order relational climate construct.

While the observed variables (items) and the four first-order factors must converge on their respective higher-order factors, these higher-order factors must also be empirically distinct in order to claim that they are capturing unique variance in their respective indicators. In sum, we hypothesize the following:

H1b: Each of the first-order factors will satisfy empirical tests of convergent and discriminant validity and therefore provide a unique and meaningful contribution to the second-order factor.

### Method

This study utilized two separate samples—one for the exploratory factor analysis (EFA) and a second for the confirmatory factor analysis (CFA). Both samples were recruited from Amazon MTurk. The inclusion criteria were the same as in Study 1 with the addition of work experience (currently working more than 20 h/week in an organization with more than 10 employees). Both surveys included three attention checks. Information on the final samples can be found in Table 3.

**TABLE 3:** Summary of samples.

	**Sample 1 (*N* = 93)**	**Sample 2 (*N* = 287)**	**Sample 3: CFA (*N* = 359)**	**Sample 4 (*N* = 354)**
Study and use	Study 1 Q-Sort	Study 2 EFA	Study 2 CFA	Study 4 Consequences
			Study 3 Antecedents	
**Demographics**				
Gender	48% male	52.3% male	64.6% male	56% male
Age (average)	22–64 years (37 years)	19–65 years (35 years)	18–63 years (31 years)	18–64 years (32 years)
Education	100% college degree	61.7% college degree	72.3% college degree	76.2% college degree
Job tenure	N/A	1–5 years	1–5 years	1–5 years
Average organization size	N/A	>100 employees	>100 employees	>100 employees

### Results

To avoid multicollinearity issues, before running the initial EFA, we had to remove some items due to high correlations. Items were removed based on the following criteria: (1) a correlation above 0.8 with another item or (2) multiple correlations above 0.75 with other items and (3) conceptual considerations. As a result of this initial screening, 12 items were removed from the item pool, leaving a total of 32 items in the first EFA. The correlations for the final items are displayed in Table 4.

**TABLE 4:** Item correlations for retained items*.

	**1**	**2**	**3**	**4**	**5**	**6**	**7**	**8**	**9**	**10**	**11**	**12**
1. V_3	1.00											
2. V_4	0.62	1.00										
3. V_7	0.64	0.71	1.00									
4. V_8	0.68	0.65	0.69	1.00								
5. V_12	0.68	0.53	0.61	0.70	1.00							
6. C_2	0.42	0.43	0.44	0.41	0.43	1.00						
7. C_4	0.53	0.46	0.46	0.46	0.48	0.74	1.00					
8. C_5	0.47	0.38	0.46	0.41	0.43	0.75	0.75	1.00				
9. C_12	0.50	0.41	0.35	0.40	0.46	0.62	0.71	0.69	1.00			
10. E_2	0.54	0.44	0.44	0.51	0.50	0.65	0.65	0.60	0.57	1.00		
11. E_4	0.55	0.47	0.46	0.51	0.54	0.59	0.59	0.53	0.53	0.72	1.00	
12. E_10	0.51	0.47	0.48	0.50	0.48	0.59	0.59	0.55	0.50	0.76	0.73	1.00

*All correlations are significant at *p* < 0.001.

The initial EFA revealed that there are four factors in the data with eigenvalues greater than 1.00. The four factors together explained 67% of variance. An examination of the rotated factor matrix revealed that some items did not load as we expected them to. Specifically, items that were intended to be part of the positive mood construct loaded on the compassion construct. Additionally, the fourth factor extracted appeared to be composed primarily of the reverse-coded items from all four constructs.

Based on this initial output, all the positive mood items were removed due to the cross loadings. The reason for removing the positive mood items rather than the compassion items was 3-fold. First, the item loadings were stronger for compassion than for positive mood. Second, the conceptual basis for the positive mood construct was weak compared to the compassion construct. Third, positive mood is partly captured through the relational energy in that relational energy captures positive energy and hence some degree of positive affect. Aside from the compassion and positive mood constructs, the two reverse-coded items from the shared vision construct that had factor loadings less that 0.40 (V9_9 and V10_R) and the two reverse-coded items from the compassion construct (C_8R ad C_9R) were also removed.

The second EFA revealed three clean factors with eigenvalues greater than 1.00. Together, the three factors explained 62% of variance. The only cross loading that was cause for concern was E_9, which was subsequently removed. The final EFA revealed three factors with all item loadings greater than 0.65 and no significant cross loadings (see Table 5).

**TABLE 5:** EFA 3 pattern matrix.

	**Factor**		
	**1**	**2**	**3**
V_3	0.74	0.08	0.04
V_4	0.77	0.02	−0.01
V_7	0.85	0.05	−0.09
V_8	0.87	−0.08	0.05
V_12	0.72	0.01	0.09
C_2	−0.06	0.80	0.10
C_4	0.05	0.86	−0.02
C_5	0.01	0.94	−0.10
C_12	0.05	0.77	−0.02
E_2	−0.01	0.27	0.66
E_4	0.07	0.08	0.74
E_10	0.01	0.07	0.82

*Extraction method: maximum likelihood with promax rotation.*

Overall, **Hypothesis 1a** was partially supported. Relational climate does appear to be a second-order latent variable, however, rather than the hypothesized four first-order factors, it appears the construct can be adequately captured by three distinct first-order factors: shared vision, compassion, and relational energy.

#### Confirmatory Factor Analysis

To test the construct validity of relational climate, CFA was used as recommended by Floyd and Widaman (1995). We tested the hypothesized model as well as three competing models. The hypothesized model demonstrated excellent fit [comparative fit index (CFI) = 0.98, Tucker–Lewis index (TLI) = 0.97, root mean square error of approximation (RMSEA) = 0.06] and superior fit to all competing models (see Table 6).

**TABLE 6:** Measurement model fit comparisons (Sample 1).

	**Competing models**				
	**Model 1: Hypothesized**	**Model 2: 1-factor**	**Model 3: 2-factor^a^**	**Model 4: 2-factor^b^**	**Model 5: 2-factor^c^**
χ^2^	135.89	512.03	260.87	357.97	400.30
*df*	52	39	64	64	64
*p*	0.00	0.00	0.00	0.00	0.00
CFI	0.98	0.85	0.94	0.90	0.89
TLI	0.97	0.82	0.92	0.88	0.87
RMSEA	0.06	0.14	0.09	0.11	0.12
SRMR	0.03	0.06	0.04	0.05	0.06

*^*a*^Model 3 combined compassion and energy. ^*b*^Model 4 combined compassion and vision. ^*c*^Model 5 combined energy and vision.*

#### Convergent Validity

The first- and second–order factor loadings and factor correlations for Model 1 are displayed in Table 7. All of the first-order item loadings were significant (*p* < 0.00) as were the second-order factor loadings (*p* < 0.00). This provides support for the convergent validity of the items and **Hypothesis 1b**.

**TABLE 7:** Model 1: Measurement model factor loadings (Sample 1).

	**Standardized estimate**	**SE**	***p*-value**	
**Factor loadings**				
*Vision to:*				
V_1	0.78	0.02	0.00	
V_2	0.68	0.03	0.00	
V_3	0.80	0.02	0.00	
V_4	0.86	0.02	0.00	
V_5	0.80	0.02	0.00	
*Compassion to:*				
C_1	0.81	0.02	0.00	
C_2	0.83	0.02	0.00	
C_3	0.82	0.02	0.00	
C_4	0.78	0.02	0.00	
*Positive energy to:*				
E_1	0.82	0.02	0.00	
E_2	0.91	0.01	0.00	
E_3	0.81	0.02	0.00	
**Second-order factor loadings**				
*Relational climate to:*				
Vision	0.83	0.03	0.00	
Compassion	0.93	0.02	0.00	
Energy	0.91	0.02	0.00	

First-order factor correlations and AVE*				

	**1**	**2**	**3**	**AVE**

1. Vision	(0.89)			0.62
2. Compassion	0.77	(0.88)		0.65
3. Energy	0.75	0.84	(0.89)	0.73

*Composite reliability in parentheses.

Overall, the first-order constructs demonstrated good discriminant validity. The shared variance between any combination of constructs was less than the average variance extracted (AVE) by each of them (Fornell and Larcker, 1981)—the only exception being the compassion–energy combination, for which the shared variance between the constructs (*r*^2^ = 0.70) was greater than the AVE by compassion (AVE_comp_ = 0.65). Despite this, there was a significant decrease in model fit when compassion and energy were paired as one construct (Δχ^2^ = 113.14, *p* = 0.00), which suggests that although the two constructs share variance, they are best modeled as two factors. Thus, **Hypothesis 1b** is supported. The final items are included in the Table 8.

**TABLE 8:** Final scale items.

**Construct**	**Item**
Shared Vision	My organization’s daily work aligns with our vision.
Shared Vision	My organization’s purpose is clear.
Shared Vision	Members of my organization have a shared purpose.
Shared Vision	My organization’s actions are guided by a shared vision.
Shared Vision	Members of my organization have similar visions of the organization’s future.
Compassion	Members of my organization are empathetic toward each other.
Compassion	People in my organization notice when others are in need.
Compassion	Members of my organization care about each other’s well-being.
Compassion	When someone in my organization is in need, my organization takes action to assist them.
Relational Energy	The relationships in my organization are a source of energy.
Relational Energy	The atmosphere in my organization is vibrant.
Relational Energy	Interactions in my organization are lively

## Study 3: Organizational Antecedents of Relational Climate

Criterion validity requires that a given construct behave in a way that is consistent with theory—that is, constructs that should theoretically predict relational climate should do so, and relational climate should lead to outcomes that are consistent with theory. Thus, the objective of this study was to begin building a nomological net for relational climate.

### Hypotheses

#### Leader–Follower Relationship

The quality of the relationship between a leader and follower is commonly captured using the leader–member exchange (LMX) framework. The LMX framework focuses on the differential quality of the relationship between leader and follower with low-quality relationships being based on the transactional part of the employment contract and high-quality relationships being based on mutual liking, trust, and respect (Graen et al., 1982).

The role of the leader in influencing work climates is well established in the literature. For example, Sims and Brinkman (2002) found that unethical work climates are shaped, and reinforced, by leader behavior. Amabile et al. (2004) found that leader behavior influences the creative climate, and Zohar (2002) found that leadership style influences the safety climate in an organization. Additionally, Schein (1985) argues that leaders act as role models for employees and, through their behavior, provide clear signals to employees of the organization’s priorities, values, and beliefs. Following this line of thinking, if an employee has a positive relationship with their leader, they are likely to emulate this type of relationship with their colleagues, and this will be reflected in the relational climate of the organization. Thus, we predict the following:

H2: Leader–member social exchange (LMSX) is positively related to perceived relational climate.

#### Procedural Justice

Procedural justice is concerned with the “fairness of the procedures used to determine outcome distributions or allocations” in organizations (Colquitt et al., 2001: 425). There is a growing body of work that suggests that procedural justice elicits emotional responses that mediate the relationship between procedural justice and organizational outcomes (Weiss et al., 1999; Murphy and Tyler, 2008). A lack of perceived fairness in an organization leads to feelings of distrust, anxiety, conflict, retribution, and retaliation (Skarlicki and Folger, 1997; Aquino et al., 2006)—all of which are known to impede the development of positive relationships (Cameron et al., 2003). Conversely, high procedural justice elicits positive emotions such as happiness and joy (Krehbiel and Cropanzano, 2000), cheerfulness (de Cremer et al., 2005), and pride (Weiss et al., 1999)—emotions associated with the facilitation of positive relationships (Fredrickson, 2001, 2004).

Finally, research suggests that perceived procedural justice also impacts a person’s sensemaking and attributions surrounding organizational decisions. For example, Krehbiel and Cropanzano (2000) found that procedural justice increases the ability of employees to derive meaning from both favorable and unfavorable decisions. Additionally, when persons perceive that they are the victim of wrongdoing, they are more likely to forgive and move on in climates perceived as having high procedural justice (Aquino et al., 2006). There is a significant body of evidence suggesting that forgiveness is important for the development and maintenance of relationships (for a review, see Enright and Fitzgibbons, 2000).

In sum, perceptions of procedural justice impact emotional, cognitive, and behavioral responses to organizational decisions, which in turn impact the relationships in the organization. Environments perceived to have high levels of procedural justice produce trust, positive emotions, and meaning, all of which facilitate positive relationships. Environments perceived to have low procedural justice elicit negative emotions, distrust, and ambiguity, all of which impede positive relationships. Thus, we expect that procedural justice will be positively related to relational climate.

H3: Procedural justice is positively related to perceived relational climate.

#### Perceived Organizational Support

Perceived organizational support (POS) refers to “employee beliefs as to the extent to which the organization values their contributions and cares about their wellbeing” (Rhoades and Eisenberger, 2002: 698). POS theory posits that, among other things, perceptions of organizational support increase an employee’s felt obligation to the organization; encourages employees to incorporate organizational membership and roles into their identity; and influences employees’ general reactions to their job (Rhoades and Eisenberger, 2002). POS also fulfills employees’ socio-emotional needs in organizations (Armeli et al., 1998; Lee and Peccei, 2007), and the fulfillment of these needs predicts relationship quality (Patrick et al., 2007). Given this, we expect POS will be positively related to relational climate. In contrast, an absence of POS would result in unfulfilled socio-emotional needs and thus a lower quality relational climate.

H4: POS is positively related to perceived relational climate.

#### Discriminant Validity of Relational Climate With Independent Variables

In order to justify the introduction and use of the relational climate construct and to meet the requirements for nomological validity, it must be shown that the construct of interest is distinct from similar constructs. If relational climate is not distinct from similar constructs, then its use does not add any value. LMSX refers specifically to the relationship an employee has with a superior, while relational climate is concerned with a person’s general assessment of their relationships at work. Procedural justice is concerned primarily with the procedures used to make organizational decisions. The reaction of the employees to those procedures is what influences the relational climate in the organization. POS is concerned with an individual’s relationship with the organization, while relational climate is concerned with the individual’s general perception of the relationships between individuals within the organization. In sum, we hypothesize the following:

H5: LMX, procedural justice, and POS are empirically distinct from perceived relational climate.

### Method

We recruited a sample from MTurk using the same criteria used in Study 2. Sample demographics can be found in Table 3 (see Sample 3 in Table 3). Structural equation modeling was used for all analyses.

#### Measures

##### Leader–member social exchange

LMSX was measured using Bernerth et al.’s (2007) eight-item measure. Sample items include “If I do something for my manager, he or she will eventually repay me” and “My relationship with my manager is composed on comparable exchanges of giving and taking.”

##### Procedural justice

Procedural justice was measured using Sweeney and McFarlin’s (1993) four-item scale. Sample items include “In your organization, how fair or unfair are the procedures used to communicate performance feedback?” and “In your organization, how fair or unfair are the procedures used to determine pay raises?”

##### Perceived organizational support

POS was measured using the short version of Eisenberger et al.’s (1986) scale. Sample items include “The organization strongly considers my goals and values” and “The organization cares about my opinion.”

#### Control Variables

##### Internal trust

We used Huff and Kelley (2003) four-item scale to measure trust. Sample items include “There is a very high level of trust throughout this organization” and “Managers in this company trust their subordinates to make good decisions.”

##### Big-five personality

Personality was used only as a control variable in this study; therefore, we used the Gosling et al. (2003) short measure of the Big-Five Personality domains. While this measure does not perform as well as the comprehensive measures of the Big-Five domains in terms of psychometric properties, it performs adequately for situations in which personality is not of prime importance (Gosling et al., 2003). Sample items include “I see myself as self-disciplined” and “I see myself as reserved.”

##### Other controls

The following demographic control variables were also collected: gender, age, nationality, and level of education. Tenure and organization size were also collected.

### Results

The correlation matrix and reliability statistics for all variables included in the structural model are displayed in Table 9. In an attempt to minimize common method variance, all variables were loaded onto a common latent factor. This technique has been used extensively in organizational research (for review, see Podsakoff et al., 2003).

**TABLE 9:** Correlation matrix for Study 3—antecedents^1^ (*N* = 359).

		**Mean**	**SD**	**1**	**2**	**3**	**4**	**5**	**6**	**7**	**8**	**9**	**10**	**11**	**12**	**13**
1	Relational climate (scale)	5.00	1.02	(0.94)												
2	LMSX	4.65	1.42	0.62	(0.97)											
3	Perceived organizational support	3.92	0.42	0.35	0.65	(0.59)										
4	Perceived organizational justice	3.41	0.92	0.65	0.65	0.68	(0.89)									
5	Internal trust	4.70	1.41	0.78	0.71	0.73	0.69	(0.93)								
6	Extraversion	2.87	0.92	0.16	0.12	0.16	0.08	0.15	(0.82)							
7	Openness	3.63	0.68	–0.02	0.03	–0.07	0.02	–0.05	0.20	(0.58)						
8	Agreeableness	3.66	0.69	0.29	0.10	0.19	0.17	0.16	0.22	0.11	(0.64)					
9	Conscientiousness	3.94	0.69	0.20	0.12	0.19	0.15	0.17	0.13	0.14	0.23	(0.70)				
10	Emotional stability	3.70	0.84	0.18	0.15	0.16	0.16	0.19	0.13	0.07	0.33	0.46	(0.82)			
11	Age	31.44	9.02	–0.06	0.09	0.05	0.02	–0.05	–0.016	–0.06	0.13	0.14	0.09			
12	Tenure	3.18	0.82	–0.03	0.09	–0.01	0.01	–0.02	–0.11	–0.11	–0.00	0.03	0.06	0.52		
13	Organization size	3.28	0.87	–0.05	0.07	–0.09	–0.02	–0.02	–0.05	–0.00	–0.03	0.07	0.04	0.23	0.17	

*^1^Cronbach’s alpha on diagonal. Correlations above 0.10 are significant at *p* < 0.05. Tenure coded as 1 = less than 6 months, 2 = 6–12 months, 3 = 1–5 years, 4 = 6–10 years, 5 = more than 10 years. Organization size coded as 1 = less than 10, 2 = 11–50, 3 = 51–100, 4 = more than 100. Age is reported in years.*

To arrive at the final model, non-significant control variables were removed sequentially, beginning with the variable with the highest *p*-value. The overall model fit was excellent [χ^2^ = 272.68, *p* = 0.00; CFI = 0.97; TLI = 0.96; RMSEA = 0.05; standardized root mean square residual (SRMR) = 0.03]. Of the control variables, only internal trust (β = 0.60, *p* = 0.00) and “agreeableness” (β = 0.15, *p* = 0.00) were significant. As hypothesized, LMSX (β = 0.11, *p* = 0.03), procedural justice (β = 0.15, *p* = 0.00), and POS (β = 0.14, *p* = 0.00) were significantly related to relational climate. Thus, support was found for Hypotheses 2–4.

#### Relational Climate Discriminant Validity With Independent Variables

The Fornell–Larcker test was used to assess the discriminant validity of relational climate with the three predictors. The shared variance between any pair of the four predictors ranged from 0.08 to 0.22 and was less than the AVE by each construct (AVE ranged from 0.61 to 0.83), which suggests that these constructs are indeed distinct (Fornell and Larcker, 1981), providing support for **Hypothesis 5**.

## Study 4: Testing a Partial Nomological Network: Consequences

The objective of Study 4 was 2-fold: (1) to replicate the CFA in an independent sample and (2) to further test the criterion validity of relational climate by testing its ability to predict specified outcomes.

### Hypotheses

#### Instrumental Ethical Climate

Instrumental ethical climate is defined as “the prevailing perceptions of employees that they need to look out for themselves and their interests, regardless of relationships with other employees, or responsibilities for the organization and its environment” (Ambrose et al., 2008: 330; see also Cullen et al., 1993). Instrumental ethical climate by definition is concerned with self-interest and gain rather than the pursuit of a shared vision that characterizes a positive relational climate. Additionally, in a recent meta-analysis (Martin and Cullen, 2006), instrumental ethical climate was found to have a strong negative correlation with caring (*r* = -0.34), which suggests that we can also expect that instrumental ethical climate would also be negatively correlated with the compassionate nature of a positive relational climate. Thus, we hypothesize the following:

H6: Perception of a positive relational climate is negatively related to perception of an instrumental ethical climate.

#### Affective Organizational Commitment

Affective organizational commitment refers to identification with, involvement in, and emotional attachment to the organization (Meyer and Allen, 1997). Employees with high affective organizational commitment remain working for an organization because they want to, rather than because they need to (continuance commitment) or because they feel they ought to (normative commitment). Employees who generate an emotional attachment to their organization generally do so because they are having an emotionally (and psychologically) satisfying experience at work (Rousseau and Aubé, 2010). Emotionally satisfying experiences require that an individual’s psychological needs are being met, including the need for belonging and relatedness (Baumeister and Leary, 1995). Given this, we would expect that a high-quality relational climate should induce affective organizational commitment.

H7: Perception of a positive relational climate is positively related to affective organizational commitment.

#### Discriminant Validity of Relational Climate With Dependent Variables

As in the previous study, in order to justify the introduction and use of the relational climate construct and to meet the requirements for nomological validity, it must be shown that relational climate is distinct from instrumental ethical climate and affective organizational commitment. Instrumental ethical climate is primarily concerned with the way in which ethical decisions are made in an organization. This is clearly distinct from relational climate, which is not concerned with decision making or ethics. Affective organizational commitment is in the same conceptual realm as relational climate in that it is concerned with emotional and psychological factors within the organization, however, the distinction between the different types of commitment individuals may have to an organization is based on their motivation for continuing to work for the organization. Relational climate is not concerned with motivation for work, rather it is interested in the experience that an employee has in the workplace with regard to their relationships and interactions.

H8: Affective organizational commitment and instrumental ethical climate are empirically distinct from relational climate.

### Method

A sample of 400 participants was recruited using Amazon MTurk. The inclusion criteria for this sample were the same as those used in Studies 2 and 3. Participants who participated in Study 2 or in Study 3 were not eligible to participate in this study to ensure that there was no overlap between the samples. The survey included three attention checks. Participants were excluded if they missed any of these attention checks. The final sample size was 354: 63% male and 36.4% female. Ages ranged from 18 to 64 years with average year in job ranging from 1 to 5 years. Structural equation modeling was used for all analyses.

#### Measures

##### Affective organizational commitment

Meyer and Allen’s (1997) scale was used to measure affective organizational commitment. Sample items include “I would be happy to spend the rest of my career with this organization” and “I really feel this organization’s problems are my own.”

##### Instrumental ethical climate

This variable was measured using a subscale from Victor and Cullen’s (1988) Ethical Climate Questionnaire. Sample items include “There is no room for one’s own personal morals or ethics in my organization” and “In my organization, people are expected to do anything to further the organization’s interests, regardless of the consequences.”

#### Control Variables

##### Value congruence

Value congruence was measured using two items proposed by Posner et al. (1985). The items are “My personal values are generally compatible with the values of my organization” and “I find that sometimes I have to compromise personal principles to conform to my organization’s expectations.”

##### Internal trust, personality, and demographic variables

This study utilized the same measures for these variables as those used in Study 3.

### Results

The model fit indices for the relational climate measurement model suggest an excellent fit (χ^2^ = 75.32, *df* = 52, *p* = 0.02; CFI = 0.99; TLI = 0.98; RMSEA = 0.04). Both the first- and second-order factor loadings were greater than 0.70 and statistically significant (*p* = 0.00) (see Table 10). This provides additional evidence of the convergent validity of the relational climate scale. The composite reliabilities of the first-order constructs were also encouraging, with all estimates greater than 0.80 (Fornell and Larcker, 1981).

**TABLE 10:** Measurement model factor loadings for Model 1A (Sample 2).

	**Std. Est.**	**SE**	***p***	
**First-order factor loadings**				
*Vision to:*				
V_1	0.78	0.02	0.00	
V_2	0.72	0.03	0.00	
V_3	0.80	0.02	0.00	
V_4	0.87	0.02	0.00	
V_5	0.81	0.02	0.00	
*Compassion to:*				
C_1	0.82	0.02	0.00	
C_2	0.76	0.03	0.00	
C_3	0.83	0.02	0.00	
C_4	0.80	0.02	0.00	
*Positive energy to:*				
E_1	0.84	0.02	0.00	
E_2	0.89	0.02	0.00	
E_3	0.83	0.02	0.00	
**Second-order factor loadings**				
*Relational climate to:*				
Vision	0.75	0.03	0.00	
Compassion	0.92	0.03	0.00	
Energy	0.86	0.03	0.00	

First-order factor correlations and AVE*				

	**1**	**2**	**3**	**AVE**

1. Vision	(0.90)			0.64
2. Compassion	0.69	(0.88)		0.65
3. Energy	0.64	0.78	(0.89)	0.73

*Composite reliability in parentheses.

Overall, the three first-order constructs demonstrated good evidence of discriminant validity. The shared variance between any combinations of constructs was less than the AVE by each of them (Fornell and Larcker, 1981). Additionally, there was a significant decrease in model fit when any two constructs were forced to be equal (Anderson and Gerbing, 1988).

The correlation matrix and reliability statistics for all variables entered into the structural model are displayed in Table 11. As in Study 3, all variables were loaded onto a common latent factor in an attempt to account for common method variances (Podsakoff et al., 1990; see also Podsakoff et al., 2003). The overall model fit was excellent (χ^2^ = 209.28, *df* = 129; CFI = 0.98; TLI = 0.97; RMSEA = 0.04). Of the control variables considered, only three were significant: value congruence predicted instrumental ethical climate (β = −0.43, *p* = 0.00) and internal trust (β = 0.15, *p* = 0.03), and tenure (β = 0.12, *p* = 0.00) predicted affective organizational commitment. As hypothesized, relational climate had a negative relationship with instrumental ethical climate (β = −0.43, *p* = 0.00), providing support for **Hypothesis 6**. **Hypothesis 7** was also supported with a positive relationship between relational climate and affective organizational commitment (β = 0.69, *p* = 0.00).

**TABLE 11:** Correlation matrix for Study 4—consequences^1^ (*N* = 354).

		**Mean**	**SD**	**1**	**2**	**3**	**4**	**5**	**6**	**7**	**8**	**9**	**10**	**11**	**12**	**13**
1	Relational climate*	5.06	0.90	(0.93)												
2	Instrumental ethical climate	3.21	0.91	–0.57	(0.84)											
3	Affective commitment	4.20	1.42	0.75	–0.57	(0.91)										
4	Value congruence	4.90	1.24	0.53	–0.64	0.51	(0.65)									
5	Trust	4.78	1.32	0.74	–0.56	0.70	0.58	(0.93)								
6	Extraversion	2.87	0.92	0.22	–0.06	0.20	0.04	0.07	(0.82)							
7	Conscientiousness	4.10	0.65	0.21	–0.14	0.15	0.16	0.11	0.20	(0.74)						
8	Emotional stability	3.86	0.77	0.23	–0.18	0.14	0.18	0.13	0.23	0.42	(0.82)					
9	Openness	3.66	0.70	0.11	–0.08	0.05	0.06	0.06	0.20	0.14	0.17	(0.64)				
10	Agreeableness	3.78	0.66	0.30	–0.24	0.24	0.23	0.26	0.17	0.27	0.29	0.09	(0.65)			
11	Age	32.53	9.65	0.06	–0.07	0.12	0.12	–0.03	0.09	0.26	0.21	0.08	0.08	1		
12	Tenure	3.29	0.79	0.03	–0.05	0.13	0.05	–0.05	0.05	0.08	0.09	–0.05	0.03	0.48	1	
13	Org. Size	3.19	0.88	–0.06	0.08	–0.01	–0.03	–0.09	–0.01	–0.05	–0.02	–0.07	–0.05	0.13	0.12	1

^1^Cronbach’s alpha in parentheses. Correlations above 0.10 are significant at *p* < 0.05. Tenure coded as 1 = less than 6 months, 2 = 6–12 months, 3 = 1–5 years, 4 = 6–10 years, 5 = more than 10 years. Organization size coded as 1 = less than 10, 2 = 11–50, 3 = 51–100, 4 = more than 100. Age is reported in years. Gender coded as 1 = male, 2 = female. *Relational climate was calculated as the composite of the scales or the second order factor.

#### Relational Climate Discriminant Validity With Dependent Variables

The Fornell–Larcker test was used to assess the discriminant validity of relational climate with the two dependent variables. The shared variance between any pair of the four predictors ranged from 0.19 to 0.77 and was less than the AVE by each construct (AVE ranged from 0.88 to 0.96), which suggests that these constructs are indeed distinct (Fornell and Larcker, 1981). Thus, **Hypothesis 8** was supported.

## Discussion and Conclusion

### Contributions

**FIGURE 1:**
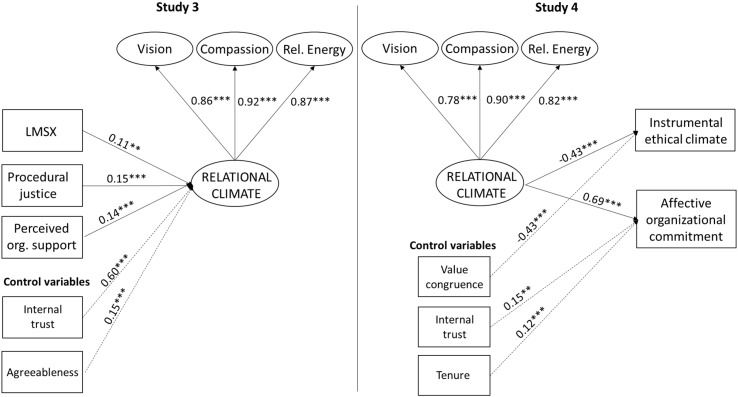
Combination findings from Study 3 and Study 4. Notes: Standardized betas are reported. ^∗∗^*p* < 0.01; ^∗∗∗^*p* < 0.001. Dotted lines represent control variables. Independent variables were allowed to covary. Only significant control variables are reported.

The key contribution of this set of studies is the development of a conceptual and operational definition of relational climate in the workplace and, based on these definitions, the construction and validation of a scale. We used a standard scale validation procedure as outlined by Hinkin (1998) to provide empirical evidence of convergent validity, discriminant validity, and reliability at both the item and first-order factor levels. Additionally, we theorized and tested a set of antecedents and consequences of relational climate in order to begin the development of the nomological network. Figure 1 provides a summary of these relationships.

### Additional Evidence of Construct Validity

We recognize that a significant limitation in the previous studies is our reliance on MTurk as a data source. Although we ensured that our samples were independent by preventing workers to participate in more than one study, questions have been raised regarding the quality of data from online participant pools (Smith et al., 2016). Due to the large number of samples required for scale development, relying on these data sources was the only practical option. However, since our initial studies, the scale has been used in a number of other unpublished studies that utilize a range of non-MTurk samples that span a number of industries. Table 12 displays the model fit statistics for the relational climate scale and summarizes the findings of each study. We include this to add further support for the psychometric properties of this scale and to show that the scale’s psychometric integrity remains across a range of samples.

**TABLE 12:** Summary of relational climate model fit statistics and findings from additional studies.

**Source**	**Sample information**		**Model fit statistics**					**Key findings**
	**Characteristics**	***N***	**χ^2^ (*df* = 51)**	**CFI**	**TLI**	**RMSEA**	**SRMR**	
Kendall, 2016	Leaders in technology United States-based technology firms	357	190.50	0.97	0.96	0.08	0.03	Relational climate predicts product innovation (*B* = 0.33, *p* < 0.05) and voice (*B* = 0.78, *p* ≤ 0.001)
Berg, 2017	Leaders in United States-based for-profit organizations	564	158.15	0.98	0.97	0.06	0.04	Relational climate predicts job engagement (*B* = 0.59, *p* < 0.001) and OCB (*B* = 0.35, *p* < 0.0001)
Alharbi, 2019	Nursing students in residency year in a large public university in Saudi Arabia	93	126.02	0.91	0.89	0.12	0.06	Relational climate predicts work engagement (*B* = 0.27, *p* < 0.001)
Leah, 2017	United States-based leaders across a range of industries	322	126.02	0.97	0.97	0.06	0.04	Relational climate predicts economic performance (*B* = 0.28, *p* < 0.001) and weakly predicts social/environmental performance (*B* = 0.10, *p* = 0.06)
Barco, 2019	College students in a large public university	1,694	422.17	0.98	0.97	0.07	0.03	Relational climate predicts student engagement (*B* = 0.89, *p* < 0.001)

*All betas are unstandardized.*

### The PNEA Scale

It should be noted that three of the four factors we proposed in this paper have been previously theorized as the Positive Negative Emotional Attractor (PNEA) scale (Boyatzis et al., 2015; Boyatzis, 2018). The PNEA scale was an initial attempt to capture the quality of relationships within intentional change theory (ICT) (Boyatzis, 2008). In ICT, resonant relationships are a critical part of shifting a person from a negative psychophysiological state [the negative emotional attractor (NEA)] to a positive psychophysiological state [the positive emotional attractor (PEA)]. This shift from the NEA to the PEA allows a person to move through the five stages of change (Boyatzis and McKee, 2005; Boyatzis et al., 2015).

In operationalizing resonant relationships in the context of ICT, Boyatzis theorized three factors: shared vision, compassion, and positive mood—together known as the PNEA scale. However, a more careful examination of the PNEA scale revealed some concerning psychometric weaknesses (Boyatzis, 2018). While the shared vision factor in the scale consistently produced strong empirical results, the items in the compassion construct confound two constructs–trust and caring–and the positive mood construct rarely loaded as expected (Boyatzis, 2018). The original items from the PNEA scale were included in our initial item pool and put through the same validation process as new items. As a result of this, none of the items in the new relational climate measure are identical to any of the PNEA items, however, some of the items in the shared vision construct are very similar with minor variations in wording.

### Limitations

While the results from this study are encouraging, our work does suffer from a number of limitations. First, although attempts were made to minimize common method bias by loading all observed variables onto a common latent factor, statistical control is no substitute for multisource data. Thus, there is likely some inaccuracy in the strength of the relationships, although not necessarily in an upward direction (Conway and Lance, 2010). On average, the common latent factor removed approximately 3% shared variance between observed variables. The use of multisource data in future studies would increase our understanding of the strength of the relationships between relational climate and its antecedents and consequences.

Second, the cross-sectional design of this study limits our ability to make claims regarding the causal direction of the hypothesized relationships to theoretical arguments. Particularly with a construct such as relational climate, which is likely to both impact and be impacted by the same variable (i.e., relational climate is likely impacted by ethical climate and also impacts ethical climate), longitudinal designs are needed to fully grasp the relationships in the nomological network. Longitudinal research designs would allow us not only to empirically untangle antecedents from consequences but also to begin to understand how relational climate emerges and fluctuates over time.

## Future Research

The most pressing area for the future is to address the limitations discussed above. As part of the continued effort to validate this measure and place it in the nomological network, future research will need to consider additionally antecedents to, and consequences of, relational climate. For example, aside from surfacing the range of benefits of positive workplace relationships (Heaphy et al., 2018), this measure could also help organizations identify and address maladaptive relational behaviors in the workplace such as bullying (Lutgen-Sandvik et al., 2007), ostracism (Ferris et al., 2008), and loneliness (Ozcelik and Barsade, 2018).

Another area of future research closely related to the extension of the nomological network is establishing relational climate as a multilevel construct (Kozlowski and Klein, 2000; Kuenzi and Schminke, 2009; Kozlowski et al., 2013). Multilevel constructs are constructs that have similar meanings at multiple levels of analysis (Chen et al., 2005). We suspect that relational climate will be particularly important at the team level of analysis, however, it may also have implications for role-based dyadic relationships (e.g., patient–doctor and coach–coachee).

Finally, as suggested by Kuenzi and Schminke (2009), there is certainly scope to examine the relationship between the different types of organizational work climates. In this study, we included the relationship between relational climate and instrumental ethical climate, however, this is just one of multiple climates conceptualized in our literature. It is important that as scholars start to reduce organizational climate and/or psychological climate into smaller facets of climate, we continue to address the discriminant and convergent validity of the different types of climates and the common and distinct antecedent and consequences among them.

## Conclusion

In conclusion, relationships are, and we believe will continue to be, a central part of organizations and organizational research. As such, it is critical that researchers have access to a measure that captures the how employees experience relationships in a given organization. This paper took the first steps toward developing a comprehensive measure of relational climate that will serve this purpose. We examined the relationship this new construct has with other established and consequential constructs in the organizational sciences. Specifically, we found that relational climate is positively associated with LMSX, procedural justice, POS, and affective organizational commitment. Additionally, we found a negative association with instrumental ethical climate. In other field data collection efforts, this scale has been used to predict product innovation, organizational citizenship behavior (OCB) voice, job engagement, economic performance, and student engagement (see Table 12 for further details on these studies). It is hoped that in the future, this measure will assist researchers in further understanding and advocating for the important role of relationships in organizational life.

## Data Availability Statement

The datasets generated for this study are available on request to the corresponding author.

## Ethics Statement

The studies involving human participants were reviewed and approved by the Institutional Review Board, Case Western Reserve University. The patients/participants provided their written informed consent to participate in this study.

## Author Contributions

RB builded the earlier test of quality of relationships. Both authors revised the original test and expanded it and wrote the manuscript. KR collected the data and analyzed it.

## Conflict of Interest

The authors declare that the research was conducted in the absence of any commercial or financial relationships that could be construed as a potential conflict of interest.
